# The G3-U70-independent tRNA recognition by human mitochondrial alanyl-tRNA synthetase

**DOI:** 10.1093/nar/gkz078

**Published:** 2019-02-14

**Authors:** Qi-Yu Zeng, Gui-Xin Peng, Guang Li, Jing-Bo Zhou, Wen-Qiang Zheng, Mei-Qin Xue, En-Duo Wang, Xiao-Long Zhou

**Affiliations:** 1 State Key Laboratory of Molecular Biology, CAS Center for Excellence in Molecular Cell Science, Shanghai Institute of Biochemistry and Cell Biology, Chinese Academy of Sciences, University of Chinese Academy of Sciences, 320 Yue Yang Road, Shanghai 200031, China; 2 School of Life Science and Technology, ShanghaiTech University, 100 Haike Road, Shanghai 201210, China

## Abstract

Alanyl-tRNA synthetases (AlaRSs) from three domains of life predominantly rely on a single wobble base pair, G3-U70, of tRNA^Ala^ as a major determinant. However, this base pair is divergent in human mitochondrial tRNA^Ala^, but instead with a translocated G5-U68. How human mitochondrial AlaRS (hmtAlaRS) recognizes tRNA^Ala^, in particular, in the acceptor stem region, remains unknown. In the present study, we found that hmtAlaRS is a monomer and recognizes mitochondrial tRNA^Ala^ in a G3-U70-independent manner, requiring several elements in the acceptor stem. In addition, we found that hmtAlaRS misactivates noncognate Gly and catalyzes strong transfer RNA (tRNA)-independent pre-transfer editing for Gly. A completely conserved residue outside of the editing active site, Arg^663^, likely functions as a tRNA translocation determinant to facilitate tRNA entry into the editing domain during editing. Finally, we investigated the effects of the severe infantile-onset cardiomyopathy-associated R592W mutation of hmtAlaRS on the canonical enzymatic activities of hmtAlaRS. Overall, our results provide fundamental information about tRNA recognition and deepen our understanding of translational quality control mechanisms by hmtAlaRS.

## INTRODUCTION

Mitochondria are the powerhouses of eukaryotic cells. One of the typical features of human mitochondria is that they harbor their own genome, encoding 22 transfer RNAs (tRNAs), 2 ribosomal RNAs and 13 proteins (1). Human mitochondria genome-encoded proteins are critical for the assembly and function for the OXPHOS complexes; thus, mitochondrial translation is an essential and fundamental event for normal mitochondrial and cellular functions (2).

Aminoacyl-tRNA synthetases (aaRSs) are a family of ubiquitously expressed enzymes, catalyzing tRNA aminoacylation to generate aminoacyl-tRNAs (aa-tRNAs) in a two-step reaction: the synthesis of an aminoacyl-adenylate (aa-AMP) and the subsequent transfer of the aminoacyl moiety to the 3′ terminus of the cognate tRNA (3,4). Aminoacylation of tRNA requires a high level of efficiency and accuracy to control the speed of aa-tRNA production and to remove mischarged tRNAs (5,6). Thus, aaRSs should precisely recognize their cognate tRNAs. In general, tRNAs always harbor identity determinants and/or anti-determinants, which facilitate the selection of the correct tRNA from a large pool of tRNA species (7).

Extensive studies have established the tRNA recognition mechanism employed by various alanyl-tRNA synthetases (AlaRSs). Initially, an Ala-inserting amber suppressor was constructed based on the substitution of the anticodon of tRNA^Ala^ GGC with CUA. This alteration did not affect Ala insertion and caused no mischarging of the tRNA^Ala^ mutant with other amino acids, suggesting that anticodon GGC in the wild-type tRNA^Ala^ was not a major determinant for alanylation (8). No contact between AlaRS and the anticodon was confirmed by RNA footprinting analysis (9). A series of mutations were introduced into this suppressor tRNA^Ala^ mutant to study the nucleotides that are crucial for aminoacylation by AlaRS. Finally, G3-U70 was identified as the major determinant for recognition by AlaRS in Ala charging. Substitution of this wobble base pair with other pairs (A3-U70, G3-C70 and U3-G70) eliminated aminoacylation with Ala both *in vitro* and *in vivo* (8–10). Conversely, transplantation of G3-U70 into other tRNAs could confer an alanylation capacity on the chimeric tRNAs (8). Consistently, G3-U70 is absolutely conserved and used as a major recognition determinant through evolution (11). In spite of G3-U70 being a recognition determinant, other elements in the acceptor helix and the various pockets of tRNA^Ala^ were also identified as important for correct interaction (12–14). Furthermore, the discriminator A73 of tRNA^Ala^ modulated the transition state of aminoacylation; however, its mutation did not impair aminoacylation (15,16). These RNA elements or structures in the acceptor stem have been considered to be an operational RNA code or the second genetic code for specific aminoacylation (8,17,18). Recently, the structural basis of the G3-U70, but not A3-U70, recognition by AlaRS was clearly provided based on *Archaeoglobus fulgidus* AlaRS (*Af*AlaRS)/tRNA^Ala^ complexes (PDB ID: 3WQY) (19,20).

**Figure 1.**
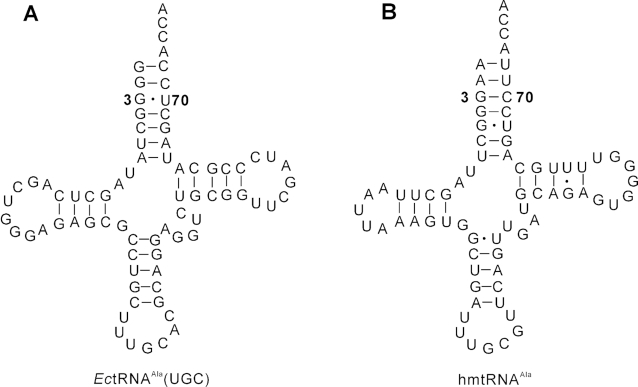
Cloverleaf structure of *Escherichia coli* tRNA^Ala^(UGC) and hmtRNA^Ala^ with the third base pair of each tRNA indicated. Sequences were obtained from the tRNA database ‘tRNAdb’ (http://trna.bioinf.uni-leipzig.de/DataOutput/).

In contrast to the absolutely conserved G3-U70 in bacterial or eukaryotic cytoplasmic tRNA^Ala^s, more divergent sequences in the acceptor stems of mitochondrial tRNA^Ala^s are observed. The mitochondrial tRNA^Ala^ from *Caenorhabditis elegans* contains the canonical G3-U70, which has been shown to be a major determinant in aminoacylation by *C. elegans* mitochondrial AlaRS (*Ce*mtAlaRS) (21). However, for the mitochondrial tRNA^Ala^ in the mitochondria of *Drosophila melanogaster*, the G-U base pair has been translocated to the second base pair (G2-U71); the third base pair is a G3-C70. The translocated G2-U71 and the G3-C70 are the major identity elements for aminoacylation by *D. melanogaster* mitochondrial AlaRS (*Dm*mtAlaRS). Interestingly, an introduced G3-U70 serves as an anti-determinant for *Dm*mtAlaRS (22). The above results showed the divergent tRNA^Ala^ recognition by various mitochondrial AlaRSs. Similarly, human mitochondrial tRNA^Ala^ (hmtRNA^Ala^) has no G3-U70 base pair (1,23), but instead has a G3-C70 Watson–Crick base pair and a translocated G5-U68 wobble base pair (Figure 1B), suggesting a different hmtRNA^Ala^ recognition mechanism by human mitochondrial AlaRS (hmtAlaRS, encoded by *AARS2*, Uniprot No. Q5JTZ9), which has not been reported.

In addition, aaRSs must also accurately recognize their cognate amino acids (5,6) Sufficient accuracy during aa-tRNA synthesis is maintained by a proofreading/editing activity of aaRSs over a selectivity threshold (24). In fact, editing activity has evolved in half of the currently identified aaRSs to remove any misactivated aa-AMPs (pre-transfer editing) and/or mischarged aa-tRNAs (post-transfer editing) (5). Correct aminoacylation of tRNA is an essential checkpoint that ensures translational fidelity. Pre-transfer editing can be further divided into tRNA-independent or tRNA-dependent pre-transfer editing, based on whether the editing occurs in the absence or presence of the cognate tRNA (25–28). Human mitochondria have 19 aaRSs, which are encoded by nuclear genes and transported into the mitochondria after synthesis in the cytoplasm (29). Interestingly, several studies have revealed that human mitochondrial aaRSs show divergence in their capacity and requirement for editing. Human mitochondrial leucyl-tRNA synthetase (hmtLeuRS, encoded by *LARS2*, Uniprot No. Q15031) or phenylalanyl-tRNA synthetase (hmtPheRS, encoded by *FARS2*, Uniprot No. O95363) has a degenerate or deleted editing domain, and shows no post-transfer editing activity *in vitro* (30–32). However, human mitochondrial threonyl-tRNA synthetase (hmtThrRS, encoded by *TARS2*, Uniprot No. Q9BW92) misactivates noncognate Ser and is active in editing of mischarged Ser-tRNA^Thr^*in vitro* (33–35).

AlaRS is responsible for producing Ala-tRNA^Ala^ (36). However, bacterial AlaRSs have been shown to misactivate noncognate Ser and Gly (37). The resultant Ser-tRNA^Ala^ and Gly-tRNA^Ala^ can be removed by the editing domain of AlaRS (38,39). In addition, Gly-tRNA^Ala^ is also removed *in trans* by a freestanding protein, D-aminoacyl-tRNA deacylase (DTD), representing a second level fidelity checkpoint in the three domains of life (40). The critical role of AlaRS-editing has been revealed in both cytoplasmic and mitochondrial systems. A slight decrease in the editing of Ser-tRNA^Ala^ by cytoplasmic AlaRS causes neurodegeneration, suggesting the critical role of editing of cytoplasmic AlaRS (41). Recently, we found that hmtAlaRS could misactivate noncognate Ser and *in vitro* hydrolyze mischarged Ser-tRNA^Ala^, whose impairment or abolition leads to embryonic lethality in mice, suggesting the critical role of editing of mitochondrial AlaRS (42). However, whether hmtAlaRS is able to misactivate noncognate Gly has not been investigated biochemically.

Mutations of human mitochondrial aaRSs or tRNAs are frequently associated with various human mitochondrial disorders. Several mutations in the *AARS2* gene have been identified in patients with clinically fatal early onset cardiomyopathies (43–45). Interestingly, *AARS2* c.1774 C>T (p.R592W) is a common founder mutation, which is carried by nearly all identified patients with the severe infantile-onset phenotype. Arg^592^ is located on the surface within the β-barrel subdomain of the editing domain of hmtAlaRS and is close to a linker for correctly positioning tRNA^Ala^ into the aminoacylation active site, based on a modeled structure. Therefore, R592W is believed to decrease the aminoacylation and editing activities of hmtAlaRS (45). However, the biochemical effect of R592W on the enzymatic activities has never been studied. Therefore, clarification of the mechanism of hmtAlaRS in alanylation is important to understand the molecular pathogenic mechanism of disease-causing hmtAlaRS and hmtRNA^Ala^ point mutations.

In the present study, we found that hmtAlaRS is a monomeric enzyme and misactivates noncognate Gly with a higher efficiency than that for Ser. HmtAlaRS catalyzes marked tRNA-independent pre-transfer editing toward Gly. Importantly, hmtAlaRS recognizes tRNA^Ala^ in a G3-U70-independent manner, with several nucleotides in the acceptor stem being critical elements. We also identified a tRNA translocation determinant from the aminoacylation domain to the editing domain during editing. Furthermore, we revealed that the R592W founder mutation leading to severe infantile-onset cardiomyopathy has no effect on tRNA aminoacylation, editing, and protein stability, suggesting another, as-yet-unidentified, disease-causing mechanism.

## MATERIALS AND METHODS

### Materials

L-Ala, L-Ser, L-Gly, NTP, GMP, tetrasodium pyrophosphate, pyrophosphatase (PPiase), Tris-base, MgCl_2_, NaCl, activated charcoal, anti-FLAG (F7425) antibodies (the FLAG tag epitope has the sequence motif N-DYKDDDDK-C), horseradish peroxidase-conjugated secondary antibodies, standard proteins (including apoferritin from horse spleen, yeast alcohol dehydrogenase and bovine serum albumin) were purchased from Sigma (St. Louis, MO, USA). [^14^C]Ala and [α-^32^P]adenosine triphosphate (ATP) were obtained from Perkin Elmer Inc. (Waltham, MA, USA). KOD-plus mutagenesis kits were obtained from TOYOBO (Osaka, Japan). Lipofectamine 2000 and 3000 transfection reagents and Dynabeads protein G were obtained from Thermo Scientific (Waltham, MA, USA). Ni^2+^-NTA Superflow resin was purchased from Qiagen Inc. (Hilden, Germany). Polyethyleneimine cellulose plates were purchased from Merck (Darmstadt, Germany).

### Cloning and expression of genes

The gene fragment encoding mature mouse mtAlaRS (Ser^26^-Leu^980^) was amplified from complementary DNA, obtained by reverse transcription-polymerase chain reaction from total RNA of mouse NIH/3T3 cell lines and was inserted between the NdeI and XhoI sites of pET30a. The open reading frame of human *AARS2* was ligated between the EcoRI and XhoI sites of pCMV-3Tag-3A to express the hmtAlaRS precursor in human cells. The construct that expresses mature hmtAlaRS, and the methods for gene expression and protein purification from *Escherichia coli* transformants have been described in a previous report (42). Protein concentrations were determined by measuring the *A_280_* of the enzyme solution. The gene encoding a hammerhead ribozyme (46) and hmtRNA^Ala^ was inserted between the PstI and EcoRI sites of pTrc99b. *In vitro* transcription and subsequent ribozyme cleavage were performed as described previously (42). Gene mutagenesis was performed according to the protocol provided with the KOD-plus mutagenesis kit. The tRNA concentration was determined by ultraviolet absorbance at 260 nm. The extinction coefficient was calculated from the sequence of each tRNA.

### ^32^P-labeling of hmtRNA^Ala^

^32^P-labeling of hmtRNA^Ala^ or its variants was performed at 37°C in a mixture containing 60 mM Tris–HCl (pH 8.0), 12 mM MgCl_2_, 15 μM hmtRNA^Ala^ or its variants, 0.5 mM dithiothreitol (DTT), 15 μM ATP, 50 μM tetrasodium pyrophosphate, 0.666 μM [α-^32^P]ATP and 10 μM *E. coli* CCA-adding enzyme (CCase) for 5 min. Finally, 0.8 U/μl of PPiase was added to the mixture for 2 min. Phenol/chloroform extraction of [^32^P]hmtRNA^Ala^ was conducted twice and the product was dissolved in 5 mM MgCl_2_.

### ATP-PPi exchange assay

The kinetics of the amino acid activation of hmtAlaRS were determined using an ATP-PPi exchange reaction in a reaction buffer containing 50 mM Tris–HCl (pH 8.0), 20 mM KCl, 10 mM MgCl_2_, 2 mM DTT, 4 mM ATP, (100–1500) mM Gly, 2 mM tetrasodium [^32^P]pyrophosphate and 200 nM enzyme at 37 °C. For the time course curve determination of hmtAlaRS and its mutants, a final concentration of 5 mM of Ala was used instead. A 9-μl aliquot of the reaction mixture was removed into 200 μl of quenching solution (2% activated charcoal, 3.5% HClO_4_ and 50 mM tetrasodium pyrophosphate) and mixed by vortexing. The solution was filtered through a Whatman GF/C filter, followed by washing with 20 ml of 10 mM tetrasodium.

### Ala acceptance assay

Equal amounts of wild-type hmtRNA^Ala^ or its various mutants (4.45 μg, final 3.6 μM) were included in an aminoacylation reaction mixture containing 50 mM Tris–HCl (pH 8.0), 20 mM KCl, 10 mM MgCl_2_, 2 mM DTT, 4 mM ATP, 50 μM [^14^C]Ala and 2 μM hmtAlaRS at 37 °C to test their Ala acceptance capacity. Samples of the reaction mixture were removed at specific time-points, quenched on Whatman filter pads and equilibrated with 5% trichloroacetic acid (TCA). The pads were washed three times for 15 min each with cold 5% TCA and then three times for 10 min each with 100% ethanol. The pads were then dried under a heat lamp. The radioactive content of the precipitates was quantified using a scintillation counter (Beckman Coulter).

### Mis-aminoacylation

Mis-aminoacylation of [^32^P]hmtRNA^Ala^ with Gly was carried out at 37 °C in a reaction mixture containing 50 mM Tris–HCl (pH 8.0), 20 mM KCl, 10 mM MgCl_2_, 2 mM DTT, 4 mM ATP, 4 μM unlabeled hmtRNA^Ala^, 0.42 μM [^32^P]hmtRNA^Ala^, 1000 mM Gly and 2 μM hmtAlaRS and its mutants. Samples at specific time points were taken for ethanol precipitation with NaAc (pH 5.2) at −20°C overnight. The precipitated samples were centrifuged (10 000 × *g*) at 4°C for 30 min, dried at room temperature for 30 min and digested with 6 μl of nuclease S1 (25 U) for 2 h at 37°C. After treatment with nuclease S1, Gly-[^32^P]hmtRNA^Ala^ should produce Gly-[^32^P]AMP and free [^32^P]hmtRNA^Ala^ should produce [^32^P]AMP. Samples (2 μl) of the digestion mixture were loaded and separated by thin layer chromatography (TLC) in 0.1 M NH_4_Ac and 5% acetic acid. The plates were visualized by phosphorimaging and the data were analyzed using Multi-Gauge Version 3.0 software (FUJIFILM, Tokyo, Japan). The amount of Gly-[^32^P]AMP produced was calculated by multiplying the total amount of hmtRNA^Ala^ (including [^32^P]hmtRNA^Ala^ and unlabeled hmtRNA^Ala^) by the relative level of charged tRNA^Ala^ in the aliquots: [Gly-[^32^P]AMP/(Gly-[^32^P]AMP + [^32^P]AMP)].

### Kinetic parameter determination of hmtRNA^Ala^ and its mutants

[^32^P]tRNA instead of [^14^C]Ala was used to more accurately determine the kinetics of the aminoacylation of hmtAlaRS for hmtRNA^Ala^ and its various mutants because of the low activity of hmtAlaRS. The kinetic parameters of hmtAlaRS for hmtRNA^Ala^ and its mutants were measured at 37°C in reaction buffer containing 50 mM Tris–HCl (pH 8.0), 20 mM KCl, 10 mM MgCl_2_, 2 mM DTT, 4 mM ATP, 1 mM Ala, ∼25 000 cpm [^32^P]hmtRNA^Ala^ or various mutants, (0.375–20.375) μM unlabeled hmtRNA^Ala^ or various mutants and 100 nM hmtAlaRS. Samples at specific time points were taken for ethanol precipitation with NaAc (pH 5.2) at −20°C overnight. Processing of the samples was similar to the procedures in the previous section.

### AMP formation

The AMP formation assay was carried out by TLC at 37°C in a reaction mixture containing 50 mM Tris–HCl (pH 8.0), 20 mM KCl, 10 mM MgCl_2_, 10 U/ml PPiase, 10 mM Ala or 1000 mM Ser or 1000 mM Gly, 3 mM [α-^32^P]ATP and 2 μM hmtAlaRS in the presence or absence of 10.7 μM hmtRNA^Ala^. Samples (1.5 μl) were quenched in 6 μl of 200 mM NaAc (pH 5.0). The quenched aliquots (1.5 μl of each sample) were spotted onto polyethyleneimine cellulose plates pre-washed with water. Separation of aminoacyl-[α-^32^P]AMP, [α-^32^P]AMP and [α-^32^P]ATP was performed in 0.1 M NH_4_Ac and 5% acetic acid. Quantification of [α-^32^P]AMP was achieved by densitometry in comparison with [α-^32^P]ATP samples of known concentrations.

### Cell culture, transfection and co-immunoprecipitation (Co-IP)

HEK293T cells were cultured in Dulbecco's modified Eagle's medium (high glucose) supplemented with 10% fetal bovine serum in a 37°C incubator with 5% CO_2_ and transfected using the Lipofectamine 2000 transfection reagent according to the manufacturer’s protocol. H9C2 cells were purchased from cell bank of our institute, cultured in the same conditions with HEK293T but transfected using Lipofectamine 3000 transfection reagent. Twenty-four hours after transfection, the cells were washed with 5 ml of ice-cold phosphate-buffered saline (PBS) twice, and lysed with 1 ml of ice-cold lysis buffer [50 mM Tris–HCl (pH 7.5), 150 mM NaCl, 5 mM ethylenediaminetetraacetic acid, 1% Triton X-100] supplemented with a protease inhibitor cocktail. The supernatant was collected using centrifugation at 12 000 × *g* for 30 min. Whole cell lysates were incubated with the anti-FLAG antibodies with agitation overnight, and then the mixture was incubated with Dynabeads protein G for 3 h. Recovered immune complexes were washed three times with ice-cold PBS plus 0.05% Tween-20 (PBST) buffer (137 mM NaCl, 2.7 mM KCl, 10 mM Na_2_HPO_4_, 2 mM KH_2_PO_4_ and 0.5‰ Triton X-100). All procedures are performed at 4°C. Proteins were eluted from the beads in 2 × protein loading buffer (100 mM Tris–HCl, 4% sodium dodecyl sulphate, 0.2% bromophenol blue, 20% glycerol and 200 mM DTT) and then subjected to western blotting.

## RESULTS

### hmtAlaRS is a monomer

**Figure 2.**
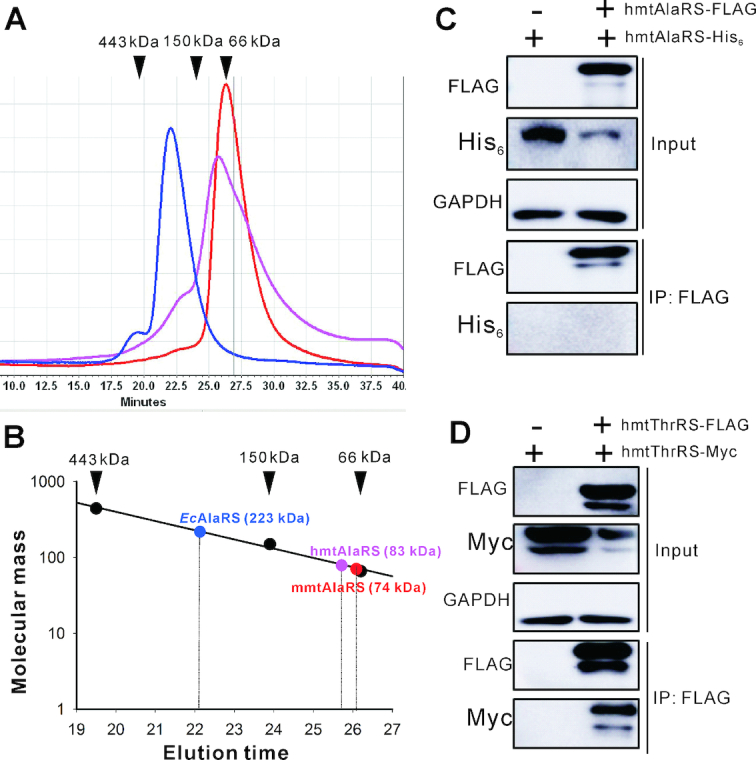
HmtAlaRS is monomeric. (**A**) Gel filtration analysis of purified *Ec*AlaRS (blue), hmtAlaRS (pink) and mmtAlaRS (red) with the elution volumes of standard proteins of known molecular weight indicated. (**B**) Apparent molecular mass determination and comparison of hmtAlaRS, mmtAlaRS and *Ec*AlaRS based on the elution volumes of the standard proteins. (**C**) After co-expression of genes encoding hmtAlaRS-His_6_ and hmtAlaRS-FLAG in HEK293T cells, hmtAlaRS-His_6_ was not precipitated by hmtAlaRS-FLAG in a Co-IP assay. (D) After co-expression of genes encoding hmtThrRS-Myc and hmtThrRS-FLAG in HEK293T cells, hmtThrRS-Myc was readily pulled down by hmtThrRS-FLAG in a Co-IP assay.

Mature hmtAlaRS (Ser^26^-Leu^986^) without the mitochondrial targeting sequence (Met^1^-Leu^25^) (47) was purified with a C-terminal His_6_-tag. The calculated molecular mass of purified hmtAlaRS together with the His_6_-tag should be 105.7 kDa. Its molecular mass was measured by gel filtration analysis based on the elution volumes of three standard proteins, apoferritin (443 kDa), yeast alcohol dehydrogenase (150 kDa) and bovine serum albumin (66 kDa). The determined molecular mass of hmtAlaRS was 83 kDa (Figure 2A and B). Considering that the AlaRS subunit is a long shaped molecule with catalytic, tRNA recognition, and editing domains connected to a globular C-terminal domain by a long helix, that the measured molecular mass would be similar, but not the same as the calculated value, is reasonable. However, the result still implied that hmtAlaRS is a monomer, like the *C. elegans* mtAlaRS (21). Using a similar method, the molecular masses of mature mouse mtAlaRS (mmtAlaRS, encoded by *Aars2*, Uniprot No. Q14CH7) (Ser^26^-Leu^980^) with a C-terminal His_6_-tag (calculated molecular mass of 107 kDa) and *E. coli* AlaRS (*Ec*AlaRS, Uniprot No. P00957, calculated molecular mass of 96 kDa) were measured, and were 74 and 223 kDa, respectively (Figure 2A and B). Obviously, mmtAlaRS should also be a monomer, while *Ec*AlaRS is not.

Furthermore, we used co-immunoprecipitation (Co-IP) to confirm that hmtAlaRS is a monomer. Our previous report used a Co-IP assay to show that hmtThrRS is a dimer (33); therefore, the enzyme could be used as a dimeric control in the present Co-IP assay. The genes encoding a C-terminal His_6_-tagged hmtAlaRS (hmtAlaRS-His_6_) and a C-terminal FLAG-tagged hmtAlaRS (hmtAlaRS-FLAG) were co-expressed in HEK293T cells. Using anti-FLAG antibodies to perform Co-IP, hmtAlaRS-His_6_ could not be precipitated with hmtAlaRS-FLAG, suggesting that hmtAlaRS-His_6_ was unable to form a homodimer with hmtAlaRS-FLAG (Figure 2C). However, when genes encoding a C-terminal Myc-tagged hmtThrRS (hmtThrRS-Myc) and a C-terminal FLAG-tagged hmtThrRS (hmtThrRS-FLAG) were co-expressed in the HEK293T cells, hmtThrRS-Myc was readily pulled down by hmtThrRS-FLAG, as expected (Figure 2D). These data further showed that hmtAlaRS is a monomer.

### The G3-U70-independent tRNA^Ala^-recognition mechanism of hmtAlaRS

The co-crystal structure of *Af*AlaRS with tRNA^Ala^ (PDB ID: 3WQY) provides valuable insights into the canonical tRNA^Ala^ recognition by AlaRS (19). The accepter stem and the G19-C56 base pair in the elbow region are two regions making extensive interactions with *Af*AlaRS (19). To elucidate the recognition of hmtRNA^Ala^ by hmtAlaRS, the following tRNA regions were selected for detailed investigation.

#### Accepter stem

**Figure 3.**
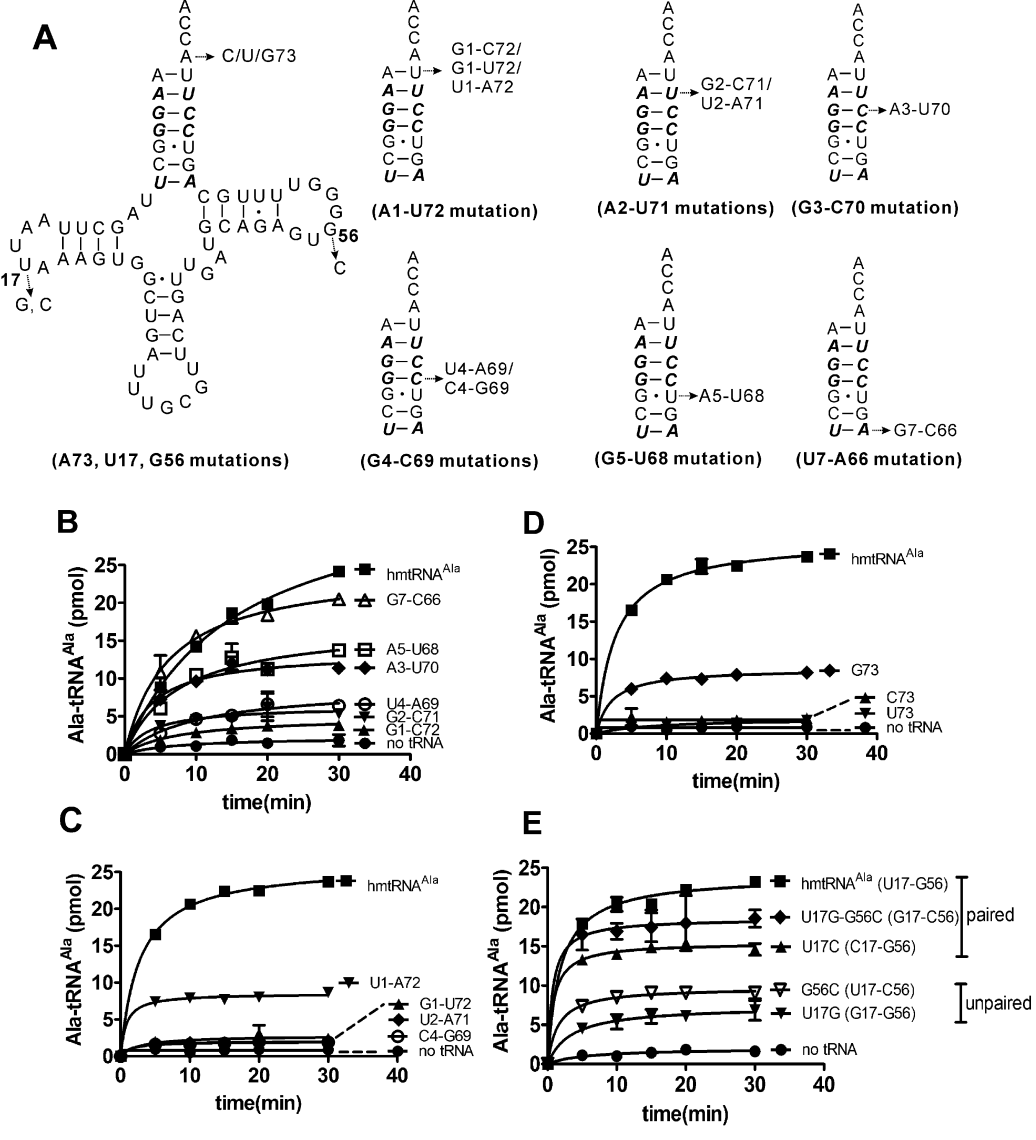
Elements for hmtRNA^Ala^ recognition. (**A**) Mutagenesis of hmtRNA^Ala^ at the acceptor stem, discriminator base and the elbow region; conserved base pairs between various mammalian mitochondrial tRNA^Ala^s were shown in bold and italic. (**B**) Ala-acceptance of tRNA mutants including G1-C72 (▴), G2-C71 (▾), A3-U70 (✦), U4-A69 (○), A5-U68 (□), G7-C66 (▵). (**C**) Ala-acceptance of tRNA mutants including U1-A72 (▾), G1-U72 (▴), U2-A71 (✦) and C4-G69 (○). (**D**) Ala-acceptance of tRNA mutants including U73 (▾), C73 (▴) and G73 (✦). (**E**) Ala-acceptance of tRNA mutants including U17C (▴), U17G (▾), G56C (▿), U17G/G56C (✦). Control in the absence (no tRNA) (•) or presence of hmtRNA^Ala^ (▪) was included in all assays. In all the graphs, mean values with error bars indicating SD are shown.

Nearly all tRNA^Ala^s harbor a conserved G3-U70 base pair, which is an essential tRNA recognition element by AlaRS, which cannot be replaced by other base pairs (8,17). However, hmtRNA^Ala^ lacks the conserved G3-U70, while possessing a Watson–Crick G3-C70 base pair and a translocated G5-U68 wobble base pair (Figure 1A and B), suggesting that the interaction between hmtAlaRS and hmtRNA^Ala^ is distinct from that between other AlaRSs and tRNA^Ala^. To study the molecular recognition mechanism of hmtAlaRS for mtRNA^Ala^, a series of mtRNA^Ala^ mutants were constructed (Figure 3A). Initially, A1-U72, A2-U71, G3-C70, G4-C69, G5-U68 and U7-A66 were mutated to G1-C72, G2-C71, A3-U70, U4-A69, A5-U68 and G7-C66, respectively (Figure 3A). C6-G67 was not mutated because of its non-conversation among different mammalian species (e.g. U6-G67 and U6-A67 in *Rattus norvegicus* and *Bos taurus*, respectively) (Supplementary Figure S1). The plateau level of Ala charging of tRNA mutants with G1-C72, G2-C71 and U4-A69 was significantly decreased while that of the mutants with A3-U70, A5-U68 and G7-C66 was only marginally affected when compared with wild-type mtRNA^Ala^, suggesting the importance of A1-U72, A2-U71 and G4-C69 in aminoacylation (Figure 3B). We further determined the kinetic parameters of hmtAlaRS for these various mutants, as shown in Table 1. The data showed that, in comparison with that for wild-type hmtRNA^Ala^, the *k*_cat_ values for G1-C72 and U4-A69 were significantly decreased, while the *K*_m_ values were only slightly affected. The *k*_cat_ and *K*_m_ values for G2-C71 were decreased and increased, respectively; however, the catalytic efficiency remained unchanged. For the translocated G5-U68, after mutation to A5-U68, the *k*_cat_ value of hmtAlaRS for the mutant was decreased and the *K*_m_ value increased moderately. Therefore, the A5-U68 mutation decreased but not abolished aminoacylation, suggesting that this base pair was important, but is not an identity element for aminoacylation. However, both the *k*_cat_ and *K*_m_ values for the other two mutants (A3-U70 and G7-C66) were not obviously changed when compared with those of wild-type hmtRNA^Ala^. These data were consistent with the Ala acceptance capacity determination and suggested that G3-C70 and U7-A66 were not important for tRNA aminoacylation.

**Table 1.** Kinetic parameters of hmtAlaRS for hmtRNA^Ala^ and its variants in aminoacylation^a^.

tRNA	*K*_m_ (μM)	*k*_cat_ (min^−1^)	*k*_cat_ / *K*_m_ (mM^−1^min^−1^)	Discrimination factor (DF)^b^
hmtRNA^Ala^	3.43 ± 0.22	4.00 ± 0.16	1.17	1
G1-C72	2.22 ± 0.19	0.31 ± 0.10	0.14	8.36
U1-A72	2.35 ± 0.77	1.41 ± 0.13	0.60	1.95
G2-C71	2.17 ± 0.67	2.43 ± 0.24	1.12	1.04
A3-U70	2.61 ± 0.45	3.31 ± 0.52	1.27	0.92
U4-A69	5.33 ± 1.47	0.56 ± 0.07	0.11	10.64
A5-U68	4.88 ± 0.09	1.38 ± 0.03	0.28	4.18
G7-C66	3.74 ± 0.64	3.94 ± 0.27	1.05	1.11
G73	3.50 ± 0.41	1.38 ± 0.09	0.39	3.00

^a^The results are the average of three independent repeats with standard deviations indicated.

^b^DF was calculated by equation DF = (*k*_cat_/*K*_m_)_hmtRNA_^Ala^/(*k*_cat_/*K*_m_)_mutant_.

Among different species (including human, mice, *R. norvegicus, B. taurus* and *Equus caballus*), A2-U71 and G4-C69 are conserved, while the first base pair is either A1-U72 or G1-U72 (Supplementary Figure S1). A1-U72 of hmtRNA^Ala^ was further changed to G1-U72 or U1-A72; and A2-U71 and G4-C69 were further mutated to U2-A71 and C4-G69, respectively (Figure 3A). The results showed that tRNAs with G1-U72, U2-A71 and C4-G69 were nearly defective in charging Ala, suggesting that the first two and the fourth base pairs are critical for aminoacylation. tRNA with U1-A72 was able to accept Ala but with an obviously decreased plateau level (Figure 3C). Indeed, kinetics determination showed that the *k*_cat_ value for U1-A72 was only 40% of that of wild-type hmtRNA^Ala^; however, the *K*_m_ value was only moderately altered (Table 1).

The above data clearly showed that hmtAlaRS recognizes hmtRNA^Ala^ in a G3-U70-independent manner, utilizing multiple nucleotide elements in the acceptor stem. Three base pairs (A1-U72, A2-U71 and G4-C69) are likely to be the most important base pairs in the acceptor stem for aminoacylation, while A5-U68 was necessary but does not function as a recognition determinant. The discrimination factor (DF), based on the relative catalytic efficiency of the mutants to wild-type hmtRNA^Ala^, was within one order of magnitude (Table 1) and the mutations at the acceptor stem mainly affected the *k*_cat_ values, which was similar to the discrimination of wild-type tRNA^Ala^ with tRNA^Ala^ mutants by *Af*AlaRS (19).

#### Discriminator base

In *E. coli* tRNA^Ala^ (*Ec*tRNA^Ala^), mutation of base A73 to U73, C73 or G73 modulates, but does not block the alanylation of these mutants (15,16). To reveal the role of A73 in the alanylation of hmtRNA^Ala^, hmtRNA^Ala^ mutants with U73, C73 or G73 were obtained and their accepting activities were assayed (Figure 3A). The results showed that alanylation of hmtRNA^Ala^ mutants with U73 or C73 was abolished, while hmtRNA^Ala^ with G73 still accepted Ala, albeit at a lower efficiency (Figure 3D), suggesting the importance of purine bases at position 73. Further kinetic parameters determination showed that the *k*_cat_ value for G73 was moderately decreased, but the *K*_m_ value did not change (Table 1). Therefore, A73 plays a crucial role in recognition by hmtAlaRS and should be a discriminator.

#### The elbow region

In hmtRNA^Ala^, U17 in the D-loop potentially forms a tertiary base pair with G56 in the TψC stem. We constructed several mutants to change the potential G56-U17 base pair: U17 was mutated to G17 to form G17-G56; G56 was mutated to C56 to produce U17-C56; U17 was mutated to C17 to form a C17-G56 Watson–Crick base pair and U17 and G56 were simultaneously changed to G17 and C56 to form the G17-C56 Watson–Crick base pair. The accepting activities of these mutants were assayed. The data showed that, compared with that of the wild-type hmtRNA^Ala^, tRNA mutants with base-paired 17–56 were able to accept Ala with only marginally decreased plateau level; while that of tRNAs with unpaired 17–56 was obviously decreased, suggesting that the potential tertiary base pair between bases 17 and 56 at the elbow region in hmtRNA^Ala^ is important for the interaction between hmtRNA^Ala^ and hmtAlaRS.

Collectively, these data clearly showed that hmtAlaRS recognizes hmtRNA^Ala^ in a G3-U70-independent manner, relying on more elements, involving at least A1-U72, A2-U71, G4-C69, A73 and the potential tertiary base pair (U17-C56) in the elbow region.

### hmtAlaRS misactivates Gly with a higher efficiency than Ser

Bacterial AlaRS [e.g. *Ec*AlaRS] is able to misactivate noncognate Ser and Gly to generate mischarged Ser-tRNA^Ala^ and Gly-tRNA^Ala^, which are then hydrolyzed via a post-transfer editing reaction by the editing domain of AlaRS (37). We have reported that hmtAlaRS misactivated Ser at a significant rate, necessitating editing for mitochondrial translational quality control (42) (Table 2). However, whether hmtAlaRS misactivates Gly is unknown. In the amino acid activation assays, the kinetic data showed that Gly was distinguished from Ala mainly via an elevated *K*_m_ (5.68 and 812.75 mM for Ala and Gly, respectively), while the *k*_cat_ values were not significantly affected, which was consistent with *Ec*AlaRS (37). The DF for Gly was 350 when compared with Ala (Table 2). Considering that the DF for Ser was 569 (Table 2), it suggested that Gly was slightly more efficiently misactivated than Ser.

**Table 2.** Kinetic parameters of hmtAlaRS for Ala, Gly and Ser in an ATP-PPi exchange reaction^a^

Amino acid	*k*_cat_ (s^−1^)	*K*_m_ (mM)	*k*_cat_ / *K*_m_ (s^−1^ mM^−1^)	DF^b^
Ala^c^	4.65 ± 0.21	5.68 ± 0.18	0.82	1
Gly	1.90 ± 0.08	812.75 ± 59.75	2.34 E-03	350
Ser^c^	0.74 ± 0.04	515 ± 50	1.44 E-03	569

^a^The results are the average of three independent repeats with standard deviations indicated.

^b^DF was calculated by equation DF = (*k*_cat_/*K*_m_)_Ala_/(*k*_cat_/*K*_m_)_Gly or Ser_.

^c^Data from (42).

### Different editing mechanism of hmtAlaRS for Gly and Ser

An AMP formation assay was further employed to elucidate the editing mechanism of hmtAlaRS for Gly. Gly was misactivated with the production of Gly-AMP and/or Gly-tRNA^Ala^. Editing of either product leads to the net consumption of ATP (yielding AMP) because of repetitive cycles of synthesis-hydrolysis of the noncognate products. This is the basis of the TLC-based AMP formation methodology in which the editing capacity is measured by monitoring the quantity of AMP produced. In the presence of tRNA, the TLC assay measures the global editing activity, including the tRNA-independent and tRNA-dependent pre-transfer editing, in addition to post-transfer editing. In the absence of tRNA, AMP is produced only from tRNA-independent pre-transfer editing activity.

**Figure 4.**
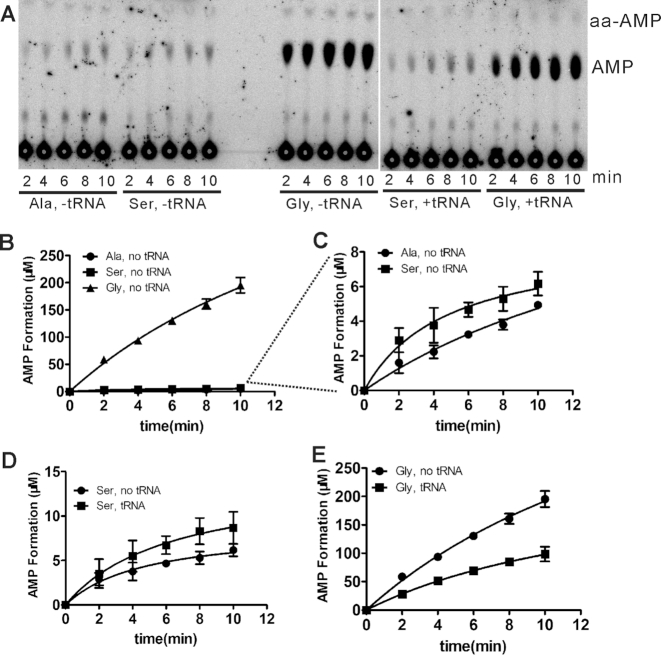
Editing mechanism of hmtAlaRS for Gly and Ser. (**A**) A representative graph showing Ala, Ser or Gly-included AMP formation of hmtAlaRS in the absence or presence of hmtRNA^Ala^. (**B**) Quantification of AMP formation by hmtAlaRS with Ala (•), Ser (▪) or Gly (▴) in the absence of tRNA; the latter two lines are further shown in (**C**) for clarity. (**D**) Editing of Ser by hmtAlaRS was enhanced in the presence (▪), compared with absence, of hmtRNA^Ala^ (•). (**E**) Editing of Gly by hmtAlaRS was inhibited in the presence (▪), compared with absence of hmtRNA^Ala^ (•). In all the graphs, mean values with error bars indicating the SD are shown.

AMP was strongly stimulated with Gly in the absence of hmtRNA^Ala^; however, little AMP was formed with Ala and Ser, clearly showing that hmtAlaRS had a strong tRNA-independent pre-transfer editing for Gly, but not Ser (Figure 4A–C). In the presence of hmtRNA^Ala^ and Ser, a little AMP was produced (Figure 4D), suggesting that hmtAlaRS mainly uses post-transfer editing to exclude Ser mischarging in spite of its low activity. Interestingly, in the presence of Gly, the amount of formed AMP was clearly lower with hmtRNA^Ala^ than that without hmtRNA^Ala^ (Figure 4E), implying that the occurrence of post-transfer editing of Gly-tRNA^Ala^ has an inhibitory effect on tRNA-independent pre-transfer editing.

Collectively, these data showed that hmtAlaRS misactivated both Gly and Ser, and displayed a much stronger tRNA-independent pre-transfer editing toward Gly than Ser.

### Arg^663^ translocates the tRNA from aminoacylation to editing domain

**Figure 5.**
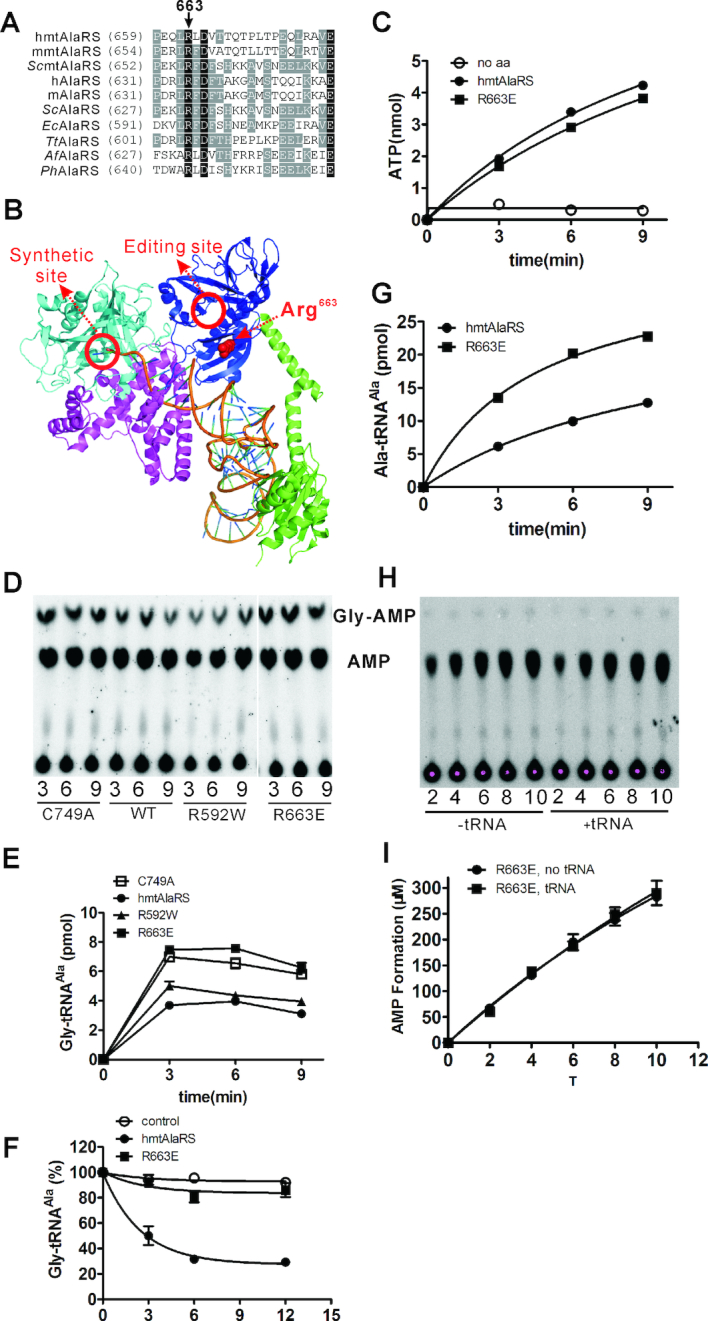
Arg^663^ is a tRNA translocation determinant. (**A**) Primary sequence alignment of Arg^663^-containing peptides from various AlaRSs. *Sc*mtAlaRS, *Saccharomyces cerevisiae* mitochondrial AlaRS (Uniprot No. P40825); hAlaRS, human cytoplasmic AlaRS (Uniprot No. P49588); mAlaRS, mouse cytoplasmic AlaRS (Uniprot No. Q8BGQ7); *Sc*AlaRS, *S. cerevisiae* cytoplasmic AlaRS (Uniprot No. P40825); *Tt*AlaRS, *Thermus thermophilus* AlaRS (Uniprot No. P74941) and *Ph*AlaRS, *Pyrococcus horikoshii* AlaRS (Uniprot No. O58035). (**B**) Location and orientation of Arg^631^ (Arg^663^ in hmtAlaRS) in the structure of the *Af*AlaRS/tRNA^Ala^ complex (PDB ID: 3WQY). Cyan, aminoacylation domain; Pink, tRNA recognition domain; Blue, editing domain; Green, C-terminal helical and globular domain. (**C**) Amino acid activation activity of hmtAlaRS (•) and R663E (▪). A control without amino acids was included (○). (**D**) A representative graph showing mischarging of hmtRNA^Ala^ with Gly by hmtAlaRS and various mutants. (**E**) Quantification of generated Gly-tRNA^Ala^ by hmtAlaRS (•), editing-defective C749A (□), R663E (▪) and R592W (▴). (**F**) Post-transfer editing of Gly-tRNA^Ala^ by hmtAlaRS (•) and R663E (▪). Spontaneous hydrolysis of Gly-tRNA^Ala^ (○) was performed without addition of the enzyme. (**G**) Aminoacylation of hmtRNA^Ala^ by hmtAlaRS (•) and R663E (▪). (**H**) A representative graph showing Gly-included AMP formation of R663E in the absence or presence of hmtRNA^Ala^. (I) Quantification of AMP formation by R663E with Gly in the absence (•) or presence (▪) of hmtRNA^Ala^. In all the graphs, mean values with error bars indicating the SD are shown.

Active sites for aminoacylation and editing are separately embedded in two domains at a distance of ∼40 Å (19,20). For AlaRS, the CCA end and the acceptor stem are dissociated from the aminoacylation domain and are then re-captured by the editing domain to orient the CCA end into the editing active site (48). The amino acid residues for binding the acceptor stem in the aminoacylation domain have been clearly determined based on the structure of *Af*AlaRS/tRNA^Ala^ (PDB ID: 3WQY) (19); however, the determinant(s) in the editing domain to orientate tRNA for editing are unknown. We found that Arg^631^ in the editing domain of *Af*AlaRS (Arg^663^ in hmtAlaRS), which is an absolutely conserved residue among bacterial, archaeal, cytoplasmic and mitochondrial AlaRSs (Figure 5A), is located in the potential entry pathway into the editing active site and points toward the acceptor stem of aminoacylation-state tRNA^Ala^, thus providing an ideal orientation platform to direct the acceptor stem into the editing active site (Figure 5B). To address this possibility, we tried to mutate the Arg^663^ of hmtAlaRS to Glu to disrupt the potential hmtAlaRS–tRNA^Ala^ interaction caused by the negative charge of Glu *versus* the positive charge of Lys. The gene encoding hmtAlaRS-R663E was overexpressed and purified in *E. coli* with similar yield to the wild-type hmtAlaRS, indicating that protein solubility was not affected (data not shown). HmtAlaRS-R663E activated Ala with similar efficiency to hmtAlaRS (Figure 5C). However, it synthesized more mischarged Gly-tRNA^Ala^ than did the wild-type hmtAlaRS, comparable with our previously constructed editing-defective C749A (42) (Figure 5D and E), supporting its defect in editing. Indeed, the result of the post-transfer editing assay showed that hmtAlaRS-R663E was unable to hydrolyze Gly-tRNA^Ala^ (Figure 5F). Based on the conservation, location, orientation and positively charged side chain (favorable for mediating the interaction with tRNA), we hypothesized that Arg^663^ is one of the determinants for directing tRNA into the editing active site during hydrolysis of mischarged tRNA^Ala^. Interestingly, the aminoacylation activity of hmtAlaRS-R663E was increased compared with that of hmtAlaRS (Figure 5G), possibly because blocking the entrance of tRNA into the editing domain facilitated faster product release.

Subsequently, Gly-included AMP formation of R663E was monitored in the absence or presence of hmtRNA^Ala^. In contrast to the wild-type hmtAlaRS (Figure 4E), the amount of formed AMP was comparable regardless of tRNA addition (Figure 5H and I). These results showed that, after eliminating post-transfer editing, tRNA addition did not further stimulate pre-transfer editing; suggesting that hmtAlaRS was unable to catalyze tRNA-dependent pre-transfer editing.

### R592W has little effect on protein stability, alanylation or tRNA mischarging of hmtAlaRS

**Figure 6.**
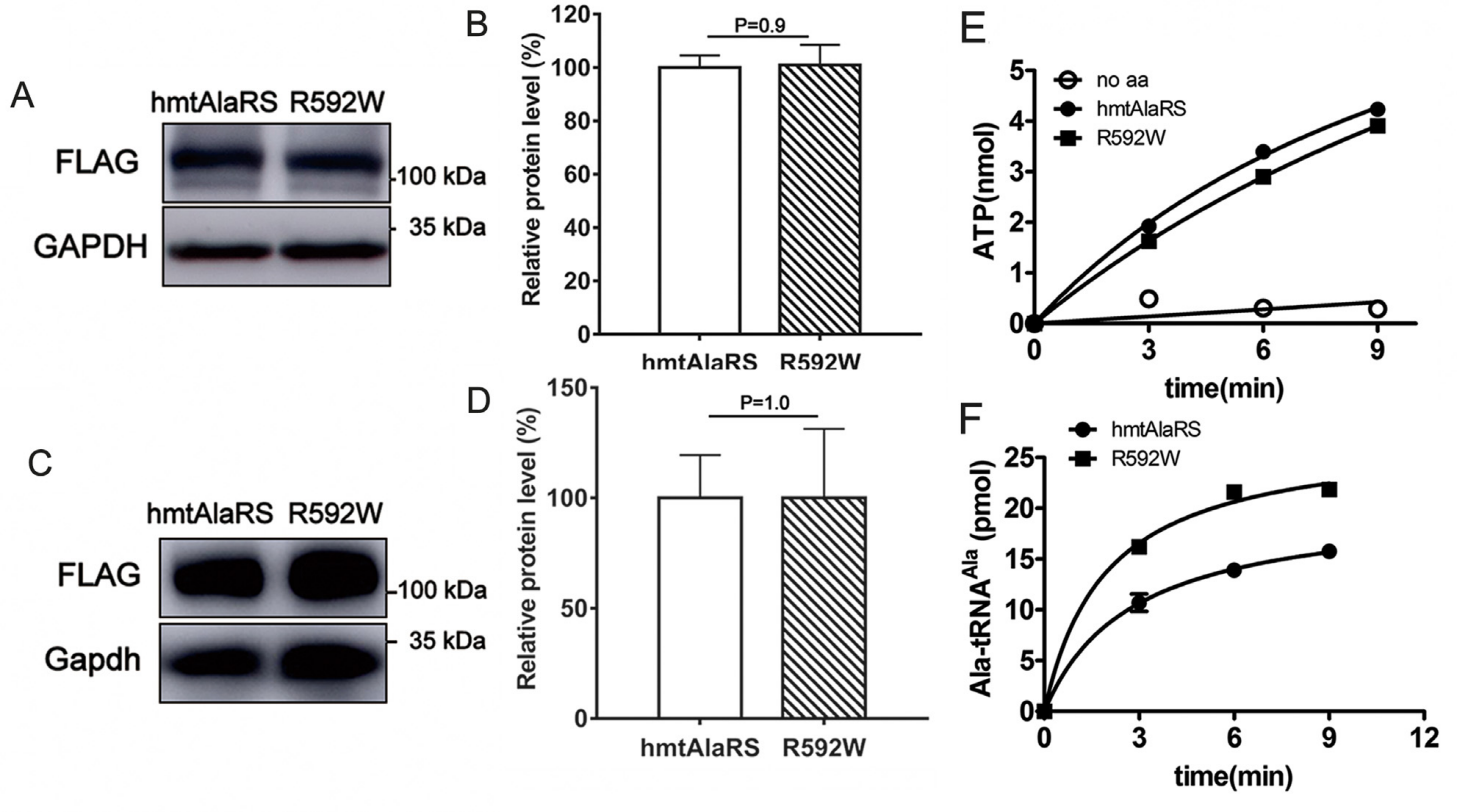
The R592W mutation has no effect on hmtAlaRS stability and enzymatic activities. Protein level determination after the expression of genes encoding hmtAlaRS and R592W in HEK293T (**A**) or H9C2 (**C**) cells. GAPDH was included as a loading control. Quantification of steady-state level of hmtAlaRS and R592W in HEK293T (**B**) or H9C2 (**D**) cells. Error bars indicated SD. (**E**) Amino acid activation activity of hmtAlaRS (•) and R592W (▪). A control without amino acids was included (○). (**F**) Aminoacylation of hmtRNA^Ala^ (10.4 μM) by 0.2 μM hmtAlaRS (•) and R663E (▪). Mean values with error bars indicating the SD are shown.

*AARS2* c.1774 C>T (p.R592W) is a common missense founder mutation, found in nearly all *AARS2*-associated clinically fatal early onset cardiomyopathies, except our two recently identified patients with cardiomyopathy with *AARS2* c.1738C>T (p.R580W) mutations (43,45,49). Despite structural modeling and suggestions of a decrease in aminoacylation and editing activities (45), the pathogenesis of R592W mutant remained unexplored. We first expressed the *AARS2* gene and the mutant gene encoding R592W in human embryonic kidney 293T (HEK293T) cells. The steady-state protein level of hmtAlaRS and R592W were comparable (Figure 6A and B), suggesting that the R592W mutation has little effect on protein stability. Considering HEK293T cells are not derived from heart tissue; the *AARS2* gene and the mutant gene encoding R592W were further expressed in a rat cardiomyocyte cell line (H9C2) and no significant alteration in protein level was also observed (Figure 6C and D). Therefore, it seems that the R592W mutation has little effect on protein stability *in vivo*, which is in contrast to our recently identified cardiomyopathy-associated R580W mutation of hmtAlaRS, which leads to structural instability. We purified R592W after overexpression of its gene in *E. coli* with similar yield to the wild-type protein, further supporting its lack of effect on protein stability (data not shown). Amino acid activation assay showed that R592W has no influence on Ala activation (Figure 6E). Charging of hmtRNA^Ala^ showed that the aminoacylation activity of R592W mutant was a little higher than the wild-type enzyme (Figure 6F). In addition, tRNA mischarging with Gly also showed that R592W accumulated similar amounts of Gly-tRNA^Ala^ compared with that of wild-type hmtAlaRS, but at lower amounts than the editing-defective C749A mutant and translocation-defective R663E mutant (Figure 5D and E). These data showed that R592W did not destroy the editing activity, similar to the R580W mutation. Therefore, the R592W mutation seems to have no effect on the canonical enzymatic activities of hmtAlaRS.

## DISCUSSION

Based on structure of *Af*AlaRS/tRNA^Ala^ (PDB ID: 3WQY), *Af*AlaRS is a homodimer (19). The C-terminal helical domain, with two long α-helices (Ala^736^ to Ala^802^ of *Af*AlaRS) mediates dimer formation. In this peptide, a series of hydrophobic residues (such as Val, Met, Leu, Ile, Pro and Phe) in both subunits form an extensive hydrophobic interaction network (20). Based on primary sequence alignment, the corresponding region in hmtAlaRS ranges from Thr^782^ to Leu^874^. Among the 21 residues in *Af*AlaRS that mediate the hydrophobic interaction, only three residues (Leu^844^, Val^848^ and Met^864^) in hmtAlaRS are conserved; the majority of residues have been changed to hydrophilic residues (such as Arg, Thr, Ser and Lys) (primary sequence alignment data not shown), which make it less likely that hydrophobic interactions are formed between subunits. Therefore, the residue divergence in hmtAlaRS likely accounts for its monomeric conformation. Indeed, *Ce*mtAlaRS has also been revealed as a monomer (21), suggesting that the monomeric state has evolved in the mitochondria of lower eukaryotes.

Our data clearly showed that, unlike other AlaRSs depending on a single G3-U70 wobble base pair in the acceptor stem of tRNA^Ala^, hmtAlaRS utilized a G3-U70-independent tRNA^Ala^ recognition mechanism. Such recognition has never been reported for the AlaRS/tRNA^Ala^ system, involving more tRNA identity elements. At the tRNA acceptor stem, it includes at least A73, A1-U72, A2-U71 and G4-C69. Mutation of G5-U68 to A5-U68 influenced aminoacylation to some extent; however, it did not eliminate hmtRNA^Ala^ charging, clearly showing that this base pair is not a major recognition determinant, in contrast to the G3-U70 in other tRNA^Ala^s. Indeed, we found that *Ec*AlaRS was unable to aminoacylate hmtRNA^Ala^ (data not shown). Intriguingly, only A2-U71, G4-C69 and A73 are absolutely conserved among various mammals; the first base pair is either A1-U72 Watson–Crick base pair (in mitochondrial tRNA^Ala^ of human and *E. caballus*) or G1-U72 wobble base pair (in mitochondrial tRNA^Ala^ of mouse, *R. norvegicus* and *B. taurus*) (Supplementary Figure S1), suggesting subtle recognition differences between hmtAlaRS and mmtAlaRS for the first base pair of the acceptor stem.

By contrast, based on the *Af*AlaRS/tRNA^Ala^ structure (PDB ID: 3WQY), the main chain of two conserved residues (Tyr^449^ and Asp^450^) and the side chain of Asp^450^ directly interact with the G3-U70 wobble base pair and the Tyr-Asp-Ser-His-Gly region (positions 449–453 of *Af*AlaRS) fitting snugly with the unique minor groove shape of G3-U70 and its adjacent base pairs (19). This peptide is always present as ‘Tyr-(Asp/Glu)-(Ser/Thr)-(His/Tyr)-Gly’ in bacterial, archaeal and eukaryotic cytoplasmic AlaRSs. However, sequence alignment showed that this peptide obviously diverged to ‘Cys-Gly-Asp-Leu-Gly’ in hmtAlaRS and ‘Ser-Gly-Asn-Leu-Gly’ in mmtAlaRS (Supplementary Figure S2A). Therefore, this divergence in the enzyme primary sequence likely explains why the G3-U70 is not conserved as a recognition determinant any more in mammalian mitochondrial tRNA^Ala^. Interestingly, in *Saccharomyces cerevisiae*, both cytoplasmic and mitochondrial AlaRSs are encoded by a single gene (*ALA1*) (50) and sequence alignment reveals that this peptide, in both cytoplasmic and mitochondrial AlaRSs, is still conserved in bacterial, archaeal and other eukaryotic cytoplasmic AlaRSs (Supplementary Figure S2A). Indeed, the third base in the acceptor stem of mitochondrial tRNA^Ala^ of *S. cerevisiae* mitochondrial tRNA^Ala^ (*Sc*mtRNA^Ala^) is maintained as G3-U70 (51) (Supplementary Figure S2B), which is likely to function as a recognition determinant. Furthermore, two separate genes encode cytoplasmic and mitochondrial AlaRSs in the lower eukaryote *C. elegans*. This peptide is present as ‘Phe-Glu-Thr-His-Gly’ in *Ce*mtAlaRS, and is very similar to bacterial, archaeal and eukaryotic cytoplasmic AlaRSs, with only the divergence of Tyr and Asp (Tyr^449^ and Asp^450^ in *Af*AlaRS) to Phe and Glu (Supplementary Figure S2A). Consistently, the third base pair in the acceptor stem of mitochondrial tRNA^Ala^ from *C. elegans* is G3-U70 (Supplementary Figure S2C) and it is indeed used as a recognition determinant (21). Therefore, the above analyses suggested that both *S. cerevisiae* mtAlaRS and *Ce*mtAlaRS recognize mitochondrial tRNA^Ala^ in a G3-U70-dependent manner and divergence in reliance on the G3-U70 in mammals is likely a later evolutionary event.

HmtAlaRS misactivates both noncognate Ser and Gly; however, it uses different editing mechanisms to remove the non-cognate amino acids. Specifically, hmtAlaRS employs very strong tRNA-independent pre-transfer editing to eliminate Gly, but not Ser. Pre-transfer editing exhausted much more ATP than post-transfer editing, as illustrated here and for other aaRSs (25–27,52), implying that Gly poses more of a threat than Ser in human mitochondria, which should be prevented at both the Gly-AMP and Gly-tRNA^Ala^ levels. However, Ser was excluded from misincorporation only at the post-transfer editing level. Besides post-transfer editing by AlaRS itself, AlaRS-produced Gly-tRNA^Ala^ is further excluded by the additional checkpoint provided by the universal DTD in bacteria, archaeal and human cytoplasm (40,53). DTD recognizes tRNA^Ala^ in a G3-U70-dependent manner (40). In human cells, DTD (encoded by the *DTD1* gene) is highly expressed in the central nervous system and is localized around the nuclear envelope region in unstressed conditions. It accumulates in the nucleus upon D-amino acid stress (54). Therefore, human DTD is possibly not localized to mitochondria. These analyses suggested that hmtAlaRS’s editing function might be the only barrier for Ala-to-Gly misincorporation, in contrast to other organisms or cell compartments. Therefore, in addition to post-transfer editing, hmtAlaRS should hydrolyze Gly-AMP in the tRNA-independent pre-transfer editing pathway, although at a much higher energy expense.

AaRSs gene mutations are frequently linked with a series of human disorders with diverse phenotypes. The pathogenic mechanisms are always distinct for each aaRS and even diverse for different mutations in a single aaRS gene, including alteration in tRNA aminoacylation, editing, localization and protein stability (2,55). Indeed, in the *AARS2* gene, R592W is a founder mutation, which is found in nearly all patients with *AARS2*-related cardiomyopathy, either homozygous or heterozygous with other mutations (43,45). Based on molecular modeling data showing exposure of Arg^592^ at the surface of the editing domain, it has been supposed that the R592W mutation decreases tRNA aminoacylation and editing activities (45). However, our biochemical data revealed that the fatal early onset cardiomyopathy-associated R592W mutation has no effect on protein stability, tRNA aminoacylation and tRNA mischarging activities of hmtAlaRS. Our recently identified cardiomyopathy-associated R580W mutation, also located at the surface of the editing domain, and has no influence on enzymatic activities; however, it obviously decreases the protein level in both cell lines and in patients (49). Therefore, we hypothesized that the R592W mutant may disturb normal mitochondrial function in a non-canonical way, such as gain-of-formation of a new protein–protein interaction network, as has been demonstrated in *GARS*-related Charcot-Marie-Tooth disease (56–58).

## Supplementary Material

Supplementary Data
