# Supplementary material for: The G3-U70-independent tRNA recognition by human mitochondrial alanyl-tRNA synthetase
Source: Nucleic Acids Res. 2019 Feb 14;47(6):3072–85. doi: 10.1093/nar/gkz078 (PMC6451123; doi:10.1093/nar/gkz078)
Supplement: Supplementary Data [file gkz078_supplemental_file.docx]

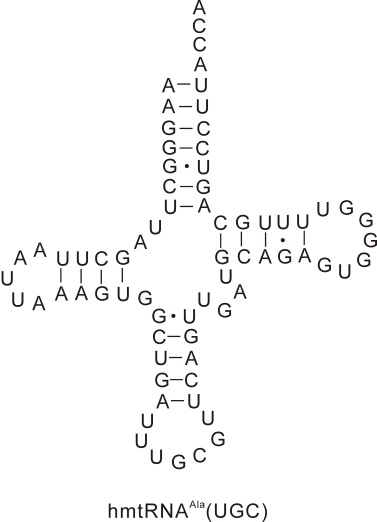

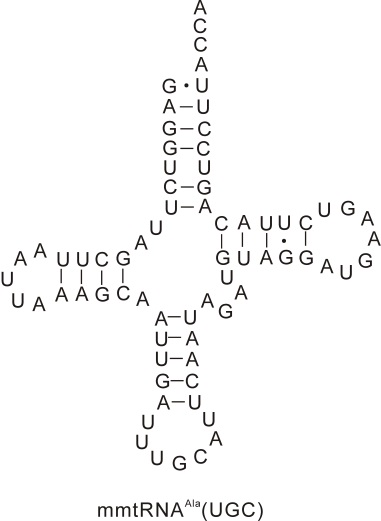

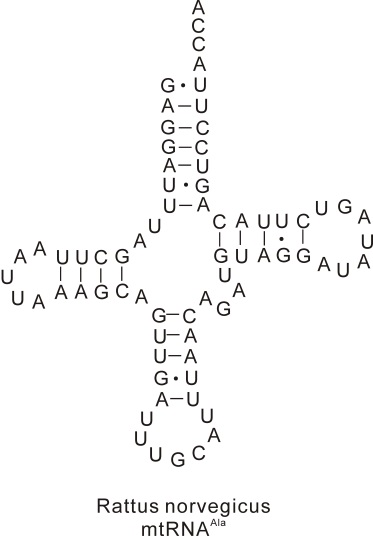


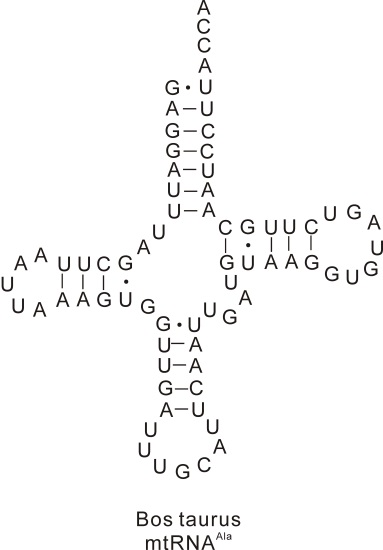

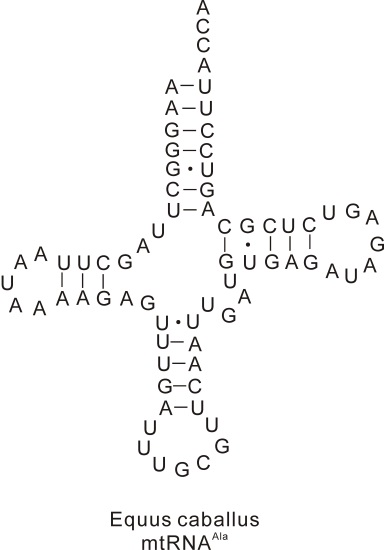


**Supplementary Figure 1**

Cloverleaf structure of mitochondrial tRNA^Ala^s from various mammals. Sequences were obtained from the tRNA database “tRNAdb” (http://trna.bioinf.uni-leipzig.de/DataOutput/).


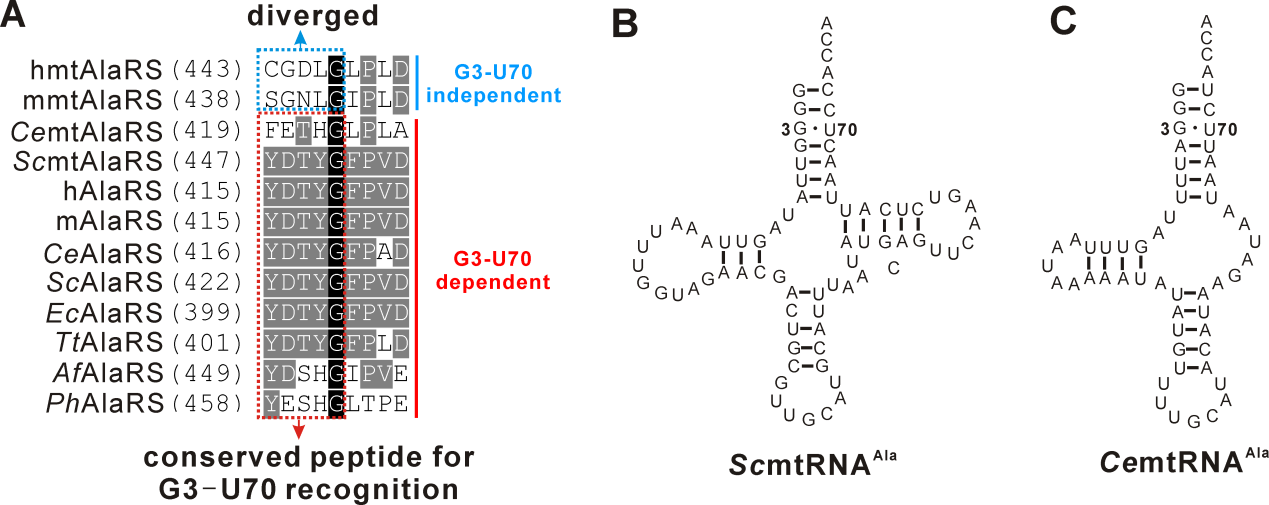


**Supplementary Figure 2. Divergence of protein elements in mammalian mtAlaRS for the recognition of G3-U70.**

(A) Primary sequence alignment of peptides for G3-U70 recognition from various AlaRSs. *Ce*mtAlaRS, *Caenorhabditis elegans* mitochondrial AlaRS (Uniprot No. Q23122); *Sc*mtAlaRS, *Saccharomyces cerevisiae* mitochondrial AlaRS; hAlaRS, human cytoplasmic AlaRS; mAlaRS, mouse cytoplasmic AlaRS; *Ce*AlaRS, *Caenorhabditis elegans* AlaRS (Uniprot No. O01541); *Sc*AlaRS, *Saccharomyces cerevisiae* cytoplasmic AlaRS; *Tt*AlaRS, *Thermus thermophilus* AlaRS; *Ph*AlaRS, *Pyrococcus horikoshii* AlaRS. Cloverleaf structures of *Saccharomyces cerevisiae* mitochondrial tRNA^Ala^ (*Sc*mtRNA^Ala^) and *Caenorhabditis elegans* mitochondrial tRNA^Ala^ (*Ce*mtRNA^Ala^) with G3-U70 highlighted are shown in (B) and (C), respectively. Sequences are obtained from the tRNA database “tRNAdb” (http://trna.bioinf.uni-leipzig.de/DataOutput/).
